# Recombinant activated factor VII as an adjunctive therapy for bleeding control in severe trauma patients with coagulopathy: subgroup analysis from two randomized trials

**DOI:** 10.1186/cc5133

**Published:** 2006-12-21

**Authors:** Sandro B Rizoli, Kenneth D Boffard, Bruno Riou, Brian Warren, Philip Iau, Yoram Kluger, Rolf Rossaint, Michael Tillinger, the NovoSeven® Trauma Study Group

**Affiliations:** 1 Department of Surgery and Critical Care Medicine, Sunnybrook Health Sciences Centre, University of Toronto, 2075 Bayview Ave., Suite H1-71, Toronto, Ontario, M4N 3M5 Canada; 2 Department of Surgery, Johannesburg Hospital, University of the Witwatersrand, 17 Pallinghurst Road, Parktown, ZA – Johannesburg 2193, South Africa; 3 Departments of Emergency Medicine and Surgery and Anesthesiology and Critical Care, Hôpital Pitié Salpêtrière, Assistance Publique-Hôpitaux de Paris, Université Pierre et Marie Curie, 4 Place Jussieu, 75005, Paris, France; 4 Department of Surgery, Tygerberg Hospital, University of Stellenboch, Fransie van Zyl Avenue, Tygerberg, Bellville 7530 Cape Town, South Africa; 5 Department of Surgery, National University Hospital, 5 Lower Kent Ridge Road, Singapore 119074; 6 Department of General Surgery B, Rambam Medical Centre, 66 Jabotinsky St., Petach Tikvah Bat-Galim, Haifa, 49100, Israel; 7 Department of Anesthesiology, University Clinics, Pauwelsstr. 30, 52057 Aachen, Germany; 8 Medical and Science Department, Novo Nordisk A/S, Novo Alle 2880 Bagsværd, Denmark

## Abstract

**Introduction:**

We conducted a post-hoc analysis on the effect of recombinant factor VIIa (rFVIIa) on coagulopathic patients from two randomized, placebo-controlled, double-blind trials of rFVIIa as an adjunctive therapy for bleeding in patients with severe trauma.

**Methods:**

Blunt and penetrating trauma patients were randomly assigned to rFVIIa (200 + 100 + 100 μg/kg) at 0, 1, and 3 hours after transfusion of 8 units of red blood cells (RBCs) or to placebo. Subjects were monitored for 48 hours post-dosing and followed for 30 days. Coagulopathy was retrospectively defined as transfusion of fresh frozen plasma (FFP) (>1 unit of FFP per 4 units of RBCs), FFP in addition to whole blood, and transfusion of platelets and/or cryoprecipitate.

**Results:**

Sixty rFVIIa-treated and 76 placebo subjects were retrospectively identified as being coagulopathic. No significant differences were noted in baseline characteristics. The rFVIIa-treated coagulopathic subgroup consumed significantly less blood product: RBC transfusion decreased by 2.6 units for the whole study population (*P *= 0.02) and by 3.5 units among patients surviving more than 48 hours (*P *< 0.001). Transfusion of FFP (1,400 versus 660 ml, *P *< 0.01), platelet (300 versus 100 ml, *P *= 0.01), and massive transfusions (29% versus 6%, *P *< 0.01) also dropped significantly. rFVIIa reduced multi-organ failure and/or acute respiratory distress syndrome in the coagulopathic patients (3% versus 20%, *P *= 0.004), whereas thromboembolic events were equally present in both groups (3% versus 4%, *P *= 1.00).

**Conclusion:**

Coagulopathic trauma patients appear to derive particular benefit from early adjunctive rFVIIa therapy.

## Introduction

Trauma is the leading cause of mortality up to the fifth decade of life [1,2] and uncontrolled hemorrhage is responsible for approximately 40% of these fatalities [2-5]. Diffuse coagulopathy is one of the most challenging situations faced by physicians treating these patients and is associated with high morbidity and mortality. Coagulopathy is common, affecting as many as 25% to 36% of trauma victims, and may develop early after injury [6,7]. It results from factors such as dilution and consumption of coagulation factors and platelets, fibrinolysis, acidosis, and hypothermia. Although coagulopathy correlates with the severity of trauma, it is also an independent risk factor of mortality [7]. There is little agreement in the contemporary literature as to the precise definition of coagulopathy in trauma (Table 1) [6-11]. Because objective measurement of coagulopathy is often unattainable in the clinical setting, current guidelines recommend empirical replacement therapy for the coagulopathic patient with diffuse microvascular bleeding [8,12]. Current management involves replacing coagulation factors (fresh frozen plasma [FFP], platelets, and cryoprecipitate) and correcting acidosis and hypothermia, steps that often are insufficient to stop the bleeding and prevent death.

Recombinant activated factor VII (rFVIIa) (NovoSeven^®^; Novo Nordisk A/S, Bagsværd, Denmark) is a hemostatic agent that acts at the site of injury to enhance thrombin generation, leading to a stable fibrin clot [13,14]. A growing number of case series and reports have described the safe and effective hemostatic properties of rFVIIa in trauma patients with uncontrolled hemorrhage refractory to conventional therapy [9,15]. These publications have described impressive results with the use of rFVIIa as a treatment option to control bleeding in high-risk, actively bleeding patients in various situations, including trauma [9,15,16], severe postpartum hemorrhage [17,18], and cardiac surgery [19-21]. Recently, our group published the first multi-center, international, randomized, placebo-controlled, double-blind study of rFVIIa in trauma and demonstrated that it is a safe and efficacious adjunctive therapy in controlling hemorrhage [22]. Considering that the majority of the patients in this study had evidence of being coagulopathic at the time of rFVIIa administration, we hypothesized that rFVIIa might have a particularly beneficial role in the treatment of diffuse coagulopathy that results from severe trauma.

To test this hypothesis, we carried out a post-hoc analysis of a subgroup of patients from the randomized prospective trial, who based on the clinical requirement for replacement therapy were identified as having coagulopathy.

## Materials and methods

The study protocol was approved by the ethics committee of each participating institution (see Appendix), and the trial was conducted according to Good Clinical Practice standards and the Helsinki Declaration. Written informed consent was obtained from all patients or, where applicable, from a legally authorized representative. Due to the emergency conditions and the possible absence of relatives at enrolment in the trial, ethics committees authorized waived informed consent. However, whenever a patient was included without written informed consent, such consent was promptly solicited from a legally authorized representative and subsequently from the patient.

The methods of the study have been previously detailed [22]. Briefly, to be eligible for inclusion, patients were to have received 6 units of red blood cells (RBCs) within a four hour period and to be of known age of at least 16 years (or legally of age according to local law) and less than 65 years. Key exclusion criteria were cardiac arrest pre-hospital or in the emergency or operating rooms prior to trial drug administration; gunshot wound to the head; Glasgow Coma Scale of less than 8 unless in the presence of a normal computed tomography scan of the head; base deficit of more than 15 mEq/l or severe acidosis with pH of less than 7.00; transfusion of 8 or more units of RBCs prior to arrival to the trauma center; and injury sustained 12 or more hours before randomization.

This was a randomized, placebo-controlled, double-blind trial with two parallel treatment arms in two separate trauma populations (blunt and penetrating traumas). Upon receiving 6 units of RBCs within a four hour period, eligible patients within each trauma population were equally randomly assigned to receive either three intravenous injections of rFVIIa (200, 100, and 100 μg/kg) or three placebo injections. The first dose of trial product was to be administered immediately after transfusion of the eighth unit of RBCs, given that the patient (in the opinion of the attending physician) would require additional transfusions. The second and third doses followed one and three hours after the first dose, respectively. Trial product was administered in addition to standard treatment for injuries and bleeding at the participating hospitals, and no restrictions were imposed on procedures deemed necessary by the attending physician, including surgical interventions, resuscitation strategies, and use of blood products. To reduce the differences in standards of care between countries and institutions, each participating trauma center had to develop specific transfusion guidelines in line with the transfusion guidelines provided in the study protocol.

### Subgroup selection

**Figure 1:**
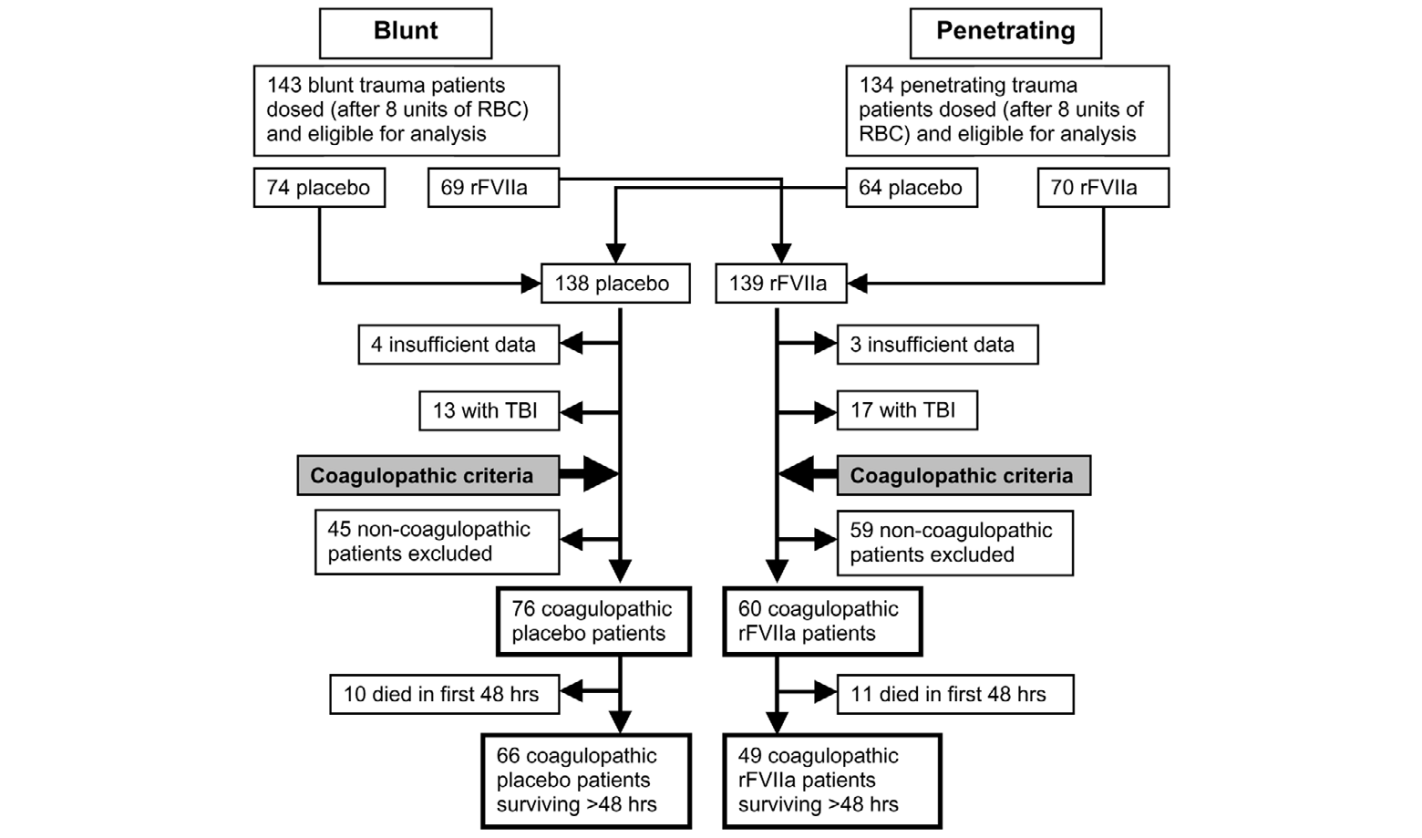
Trial profile. The figure shows the number of penetrating and blunt trauma patients eligible in the two groups (placebo and recombinant activated factor VII [rFVIIa]), the exclusion of patients because of traumatic brain injury (TBI) and insufficient data concerning the coagulopathic state, and the number of patients finally adjudicated to the placebo and rFVIIa analysis. RBC, red blood cells.

Patients included in the two arms of the trial (blunt and penetrating traumas) were pooled together. Because it was not possible to objectively determine a patient's coagulopathic status at study entry (due to the lack of a consensus laboratory definition for coagulopathy, time constraints, and limitations of laboratory testing in a trauma setting), a post-hoc state of coagulopathy was defined based on current transfusion guidelines [8]. For the purpose of the present analysis, coagulopathic patients were identified using the following definition: an ongoing bleeding that required the use of transfusion with FFP and RBC units at a ratio of 1 or more units of FFP for every 4 units of RBCs, and/or the use of FFP with whole blood, and/or transfusion of platelets, and/or the transfusion of cryoprecipitate. This definition was used because no consensus definition currently exists that adequately defines coagulopathy. Because traumatic brain injury (TBI) mandates a different fluid and transfusion management, carries a higher risk for coagulopathy, and has outcomes that are distinctive from other polytrauma patients, patients with TBI were also excluded from this analysis [23]. A flowchart of the present study is depicted in Figure 1.

### Endpoints

The primary endpoint was the number of RBC units (allogeneic RBCs, autologous RBCs, and whole blood) transfused during the 48-hour period after the first dose of trial product, as previously described [22]. Outcome of therapy was further assessed through requirement for other transfusion products, massive transfusion (defined as more than 20 units of RBCs inclusive of the 8 pre-dose units), time on ventilator, time in the intensive care unit (ICU), and serious adverse events, including the predefined critical complications of multiple organ failure (MOF), acute respiratory distress syndrome (ARDS), and death, all recorded until day 30. Because mortality is not a sensitive variable in a trauma population, we also studied a composite endpoint that comprised death, MOF, and ARDS, repeating the analysis performed in the original randomized controlled trial (RCT) [24,25]. To enable us to compare the durations of hospitalization and of ICU admission while taking into account mortality rates, we calculated the number of hospital-free days and ICU-free days within one month after trauma, with all deceased patients being given a score of 0 hospital- or ICU-free days, as previously described [26].

Separate analyses were performed in which patients who died within 48 hours were excluded, as previously described [22]. Patients who died within 48 hours were excluded because in a large proportion of these patients, care was futile and 48-hour transfusion requirements could not be objectively assessed for patients who were alive for only a few hours.

### Statistical analysis

Data are expressed as mean ± standard deviation or as median and range. All statistical comparisons were two-tailed, and *P *less than 0.05 was considered significant. Comparison between the two groups was performed using the Wilcoxon-Mann-Whitney rank sum tests for continuous outcomes (transfusions, time on ventilator, and time in ICU), and Fisher's exact test was used for binary outcomes (massive transfusion, MOF, ARDS, and mortality). The differences between groups were estimated by the Hodges-Lehman shift with 95% confidence interval (CI) for continuous outcomes, and relative risk reductions (RRR) and numbers needed to treat (NNT) with 95% CIs were used for the binary outcomes. Patients who died within 48 hours were assigned highest rank (worst outcome) in the Mann-Whitney test and in the Hodges-Lehman estimation.

## Results

Of the 277 randomly assigned patients eligible for analysis, 30 were excluded from evaluation because of TBI and seven others for insufficient data on their coagulopathic status. Thus, using the defined criteria for coagulopathy, the subgroup of coagulopathic patients comprised a total of 136 patients, 76 of whom had received placebo and 60 of whom had received rFVIIa (Figure 1). A comparative overview of baseline characteristics of the selected coagulopathic patient subgroup, according to the defined criteria, versus the non-coagulopathic patients is depicted in Table 2.

Baseline characteristics were broadly concordant between non-coagulopathic and coagulopathic patients, with the exception of significant differences in the use of FFP, platelets, and cryoprecipitate (the basis for the definition of coagulopathy) and differences in hematocrit, prothrombin time (PT) and pH (also reflective of coagulopathy), and injury severity score (Table 2). Of note, the two groups had similar baseline laboratorial coagulation profiles, including activated partial thromboplastin time (aPTT), PT, platelet count, and fibrinogen levels. Table 2 also provides data on the use of FFP, platelets, and cryoprecipitate from time of administration of trial drug to 48 hours.

The remaining analysis will focus exclusively on the coagulopathic patients. The baseline characteristics of injury severity score and physiological and coagulation variables were comparable between the placebo- and rFVIIa-treated coagulopathic patient groups, with no significant differences between groups (Table 3).

**Figure 2:**
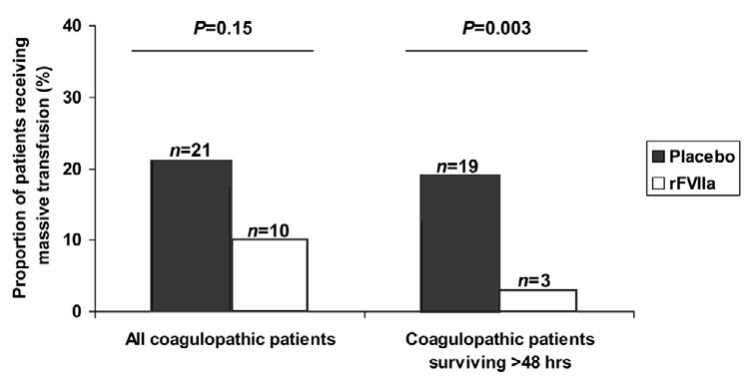
Comparison of the proportion of patients (all coagulopathic patients as well as coagulopathic patients surviving more than 48 hours) requiring massive transfusion. Massive transfusion was defined as more than 12 red blood cell (RBC) units within 48 hours of the first dose, which equals more than 20 units of RBCs (inclusive of the 8 pre-dose units) in the placebo and recombinant activated factor VII (rFVIIa) groups.

rFVIIa significantly reduced 48-hour RBC requirements by 2.6 units (all patients) and 3.5 units (48-hour survivors) compared with placebo (Table 4). Irrespective of whether the analysis included all or only 48-hour survivors, the need for FFP was also significantly reduced by rFVIIa treatment whereas platelet requirement was significantly reduced only among the 48-hour survivors (Table 4). Massive transfusion was found to be significantly reduced in patients surviving more than 48 hours who received rFVIIa as compared with placebo (*P *= 0.003, RRR 79%, 95% CI 32% to 93%) (Figure 2) but not when all patients were included. Treatment with rFVIIa was associated with an even greater reduction in exposure to RBCs, FFP, and platelets in patients surviving more than 48 hours (Table 4). In these patients, all blood-product requirements were more than halved in the rFVIIa group.

The summation of the baseline characteristics (including causes of death) of the coagulopathic patients who died early (<48 hours) is presented in Table 5. Placebo and rFVIIa groups were similar in all aspects except for a higher prevalence of penetrating injuries among those treated with rFVIIa. Sixteen of the 21 early deaths (76%) were due to exsanguination, indicating that failure to halt bleeding in patients transfused with 8 units of RBCs in the first hours is often fatal. Of note, the patients who died early had a markedly prolonged aPTT/PT but not as abnormal a platelet count or fibrinogen level.

Results for investigator-reported clinical complications and mortality within 30 days are summarized in Table 6. Treatment with rFVIIa significantly reduced the incidence of ARDS among all coagulopathic patients (*P *= 0.04, RRR 86%, 95% CI 0% to 98%, NNT 9.83, 95% CI NNT 5.15 to 88.25) and among patients surviving more than 48 hours (*P *= 0.04, RRR 85%, 95% CI 0% to 98%). rFVIIa also significantly reduced the combined endpoint of MOF and/or ARDS among all coagulopathic patients (*P *= 0.004, RRR 83%, 95% CI 29% to 96%, NNT 6.10, 95% CI NNT 3.71 to 18.23) and among patients surviving more than 48 hours (*P *= 0.004, RRR 90%, 95% CI 23% to 99%) (Table 6).

## Discussion

In trauma patients with coagulopathic hemorrhage, rFVIIa is emerging as a potentially valuable new damage-control tool for use when hemorrhage is refractory to standard-of-care treatment [9,15,22,27-30]. The analysis reported here is based on data from two randomized parallel controlled trials in severely injured patients [22]. In these trials, rFVIIa reduced the need for blood transfusion in blunt trauma patients surviving for 48 hours and with a trend toward less RBC transfusion in patients with penetrating trauma. At study entry, it was not possible to identify this coagulopathic subgroup of patients, because available routine laboratory testing (aPTT, PT, platelet count, and fibrinogen levels) did not accurately identify clinically significant coagulopathic bleeding. The platelet count does not reflect platelet-impaired function such as in hypothermia and acidosis, typically present in the massively bleeding trauma victim [31]. PT and aPTT are poor predictors of clinical bleeding unless a marked prolongation (that is, prolongation beyond the limit of machine measurements) is observed [30]. Because blood samples are typically re-warmed to 37°C, there is an underestimation of coagulopathy in hypothermic conditions typical of critical trauma patients [31]. In addition, the time required for these routine tests as well as for a more thorough analysis of coagulation state to be performed – approximately 45 minutes – precludes an accurate 'real time' reflection because the patients are usually massively transfused and infused during the time period elapsing between sampling to results [12]. Although there are no universally accepted laboratory standards for the definition of coagulopathy in these severely traumatized patients (see Table 1 for a summary of differing definitions found in the trauma literature), the clinical diagnosis of coagulopathy is familiar to most intensivists and surgeons. This is characterized by the clinical assessment of ongoing bleeding and the observation of oozing from cut surfaces, intravascular catheter sites, or mucus membranes [31].

Our post-hoc analyses identified patients who were believed by their treating physicians to have clinically significant coagulopathic bleeding, as reflected by the empirical replacement therapy that was administered. In this group, treatment with rFVIIa significantly reduced the need for all blood products, surrogate markers of bleeding, as well as potential donor exposure as compared with placebo during standard-of-care management of hemorrhage (Table 4). Furthermore, in these coagulopathic patients, early adjunctive treatment with rFVIIa had a significant effect on the risk for developing MOF and ARDS in the 30 days after traumatic injury (Table 5).

Our analyses of data also identified a significant effect of rFVIIa treatment on patients' requirement for massive RBC transfusion in the first 48 hours of the study in patients surviving more than 48 hours. Approximately 29% of coagulopathic patients in the placebo group required an additional 12 units of RBC transfusion (to the initial 8 units prior to trial drug) to manage ongoing hemorrhage as compared with a much-reduced need for massive transfusion in the rFVIIa-treated coagulopathic group, in which only 6% of patients required this level of transfusion (Figure 2). Massive transfusion of this type – 20 units of RBCs administered from time of injury to assessment at 48 hours after study entry – represents ongoing hemorrhage equivalent to at least two blood volumes [32,33]. The effect of rFVIIa to arrest such massive blood loss reduces patient exposure to excessive transfusion of blood products and inherent risk factors for immunological and infectious risks. It is known that with increasing transfusion requirements, there is a greater risk of morbidity and potentially fatal complications associated with traumatic hemorrhage. This may be due in part to a correlation between the severity of trauma and transfusion needs but also in part to the negative effects of transfusions *per se*. Indeed, the risk of developing ARDS and MOF after trauma has been shown to correlate with increasing transfusion requirements [34,35]. Our analysis of data from coagulopathic patients treated with adjunctive rFVIIa revealed a significant improvement in clinical outcome as compared with placebo. Significant reductions in the combined endpoint of ARDS and/or MOF were seen in rFVIIa-treated subjects at day 30, when these complications occurred in just 3% of patients as compared with a rate for ARDS and/or MOF of one out of every five placebo-treated coagulopathic subjects (Table 5).

RFVIIa significantly reduced the incidence of ARDS among all patients. Considering only the patients surviving more than 48 hours, ARDS (2%) or the combined endpoint of ARDS and/or MOF (2%) and the combined endpoint of ARDS/MOF/death (6%) were also significantly reduced up to day 30 in the group that received rFVIIa treatment, in contrast to a 14% rate of ARDS, a 20% rate of ARDS or MOF, and a 23% rate of death, MOF, or ARDS in placebo-treated subjects over the same follow-up period. Within this subgroup, it was shown that treatment with rFVIIa was particularly beneficial in effecting a reduction in the primary clinical endpoint of RBC requirements during 48 hours (Table 4). We noted a 3.5-unit reduction in RBC transfusion associated with treatment. The reductions in RBC requirement in this subgroup were accompanied by a similar reduction in FFP of 800 ml and reduced platelet requirements of 50 ml, in essence halving transfusion requirements (Table 4).

This study was designed with the intent of being hypothesis-generating and has limitations that deserve comment. It is a post-hoc analysis of the only RCT on the use of rFVIIa in trauma to date. The original RCT has pitfalls, some of which are inherent to clinical trials designed to study severely traumatized and actively bleeding patients during the earliest and most acute phase of resuscitation. One limitation was the difficulty of establishing uniform standards of care among 23 different institutions spread across eight countries. The original trial elected to use blood transfusion as a surrogate marker of bleeding, a widely accepted but still imperfect marker. Whereas the original RCT analyzed blunt and penetrating trauma patients separately, considering the smaller patient sample of coagulopathic patients available, the present study analyzed all coagulopathic patients combined, irrespective of the mechanism of injury. The definition of coagulopathy used is limited and imperfect because it permits a patient diagnosed as coagulopathic but for any reason not treated with blood products to be excluded from this analysis. In addition, the expectation was that coagulopathy secondary to either blunt or penetrating trauma would equally respond (or not) to the intervention. The present study also analysed the data both including all patients and excluding those who died early, thus repeating the analysis performed in the original RCT.

Despite the limitations, the findings of our post-hoc analysis of a subgroup of patients support the premise that rFVIIa is a valuable addition to the therapeutic armamentarium in the management of severe trauma, helping to arrest life-threatening coagulopathic hemorrhage, reducing transfusion requirements and associated risks, and having the potential to impact on the longer term morbidity and mortality associated with traumatic bleeding [9,15,22,27,28,36]. Moreover, our analysis identified clinically coagulopathic patients as the high-risk group of trauma patients who might benefit the most from rFVIIa therapy. Considering the limited therapeutic options and the high mortality associated with post-traumatic diffuse coagulopathy, this study supports the validity of considering the use of rFVIIa in the management of these patients. However, the hypothesis generated by this post-hoc analysis requires corroboration from other studies before any definitive recommendation can be advised.

## Conclusion

This post-hoc analysis supports the assertion that coagulopathy is common in patients with severe trauma. Of the 277 trauma patients included in a clinical trial after receiving 8 units of RBCs, 49% were identified as coagulopathic (136 patients). There is, however, little agreement on a diagnosis for traumatic coagulopathy or on its objective measurement.

The results of this study suggest that the subgroup of coagulopathic trauma patients receive particular benefit from rFVIIa therapy. Compared to a control group, coagulopathic patients treated with rFVIIa required significantly less blood transfusion with reductions in RBCs, FFP, platelets, and massive transfusions. The use of rFVIIa led to a reduction in ARDS and/or MOF and did not increase thromboembolic complications. Considering the mortality and limited therapeutic options, this study supports the concept of considering rFVIIa for the management of trauma patients with coagulopathy.

## Key messages

• Coagulopathy is common in severe trauma and lacks objective definition.

• Administration of rFVIIa to coagulopathic trauma patients significantly reduces the need for blood and blood-product transfusion.

• Administration of rFVIIa to coagulopathic trauma patients reduces the incidence of ARDS and/or MOF without increasing thromboembolic complications.

• Coagulopathic trauma patients appear to benefit from rFVIIa therapy.

## Abbreviations

aPTT = activated partial thromboplastin time; ARDS = acute respiratory distress syndrome; CI = confidence interval; FFP = fresh frozen plasma; ICU = intensive care unit; MOF = multiple organ failure; NNT = numbers needed to treat; PT = prothrombin time; RBC = red blood cell; RCT = randomized controlled trial; rFVIIa = recombinant activated factor VII; RRR = relative risk reductions; TBI = traumatic brain injury.

## Competing interests

SBR, KDB, YK, RR, and BR have received lecture and/or consultancy fees from Novo Nordisk A/S. RR has received lecture sponsorship from Novo Nordisk A/S. SBR is a member of the Scientific International Advisory Board for rFVIIa. Novo Nordisk A/S is financing the article-processing charge. MT is an employee of Novo Nordisk A/S. BW and PI declare that they have no competing interests.

## Authors' contributions

All authors made substantive intellectual contributions to the making of this manuscript and have given final approval of the version to be published. SBR, KDB, BR, BW, PI, YK, and RR were co-principal investigators in the original RCT. SBR, KDB, BR, BW, PI, YK, RR, and MT made substantial contributions to the conception and design of the study and to the analysis and interpretation of data and were involved in drafting the manuscript and revising it critically. All authors read and approved the final manuscript.

## Appendix

Data and safety monitoring board: Howard Champion, Annapolis, MD, USA (chairman); Abe Fingerhut Paris, France; Richard Weiskopf, San Francisco, CA, USA; Miguel A Escobar, Houston, TX, USA. *Ad hoc *member: Torben Sörensen (statistician), StatCon Aps, Alleroed, Denmark.

Sponsor: Novo Nordisk A/S, Bagsværd, Denmark.

Statistician: Tine Sörensen, MSc, Novo Nordisk A/S, Bagsværd, Denmark.

Investigators of the NovoSeven^® ^Trauma Study Group and Trial centers: South Africa: KD Boffard, Johannesburg General Hospital, Johannesburg; BL Warren, Tygerberg Hospital, Cape Town; A Nicol, Groote Schuur Hospital, Cape Town; R Tracey, Unitas Hospital, Centurion; JSS Marx, Pretoria Academic Hospital, Pretoria; E Degiannis, Chris Hani Baragwanath Hospital, Johannesburg; J Goosen, Milpark Hospital, Johannesburg; F Plani, Union Hospital, Alberton; LM Fingleson, Sunninghill Hospital, Sandton. France: B Riou, Hôpital Pitié Salpêtrière, Paris; JF Payen de La Garanderie, Hôpital Michallon, Grenoble; J Marty, Hôpital Beaujon, Clichy; R Krivosic-Horber, Hôpital Roger Salengro, Lille; M Freysz, Hôpital Général, Dijon; JE de La Coussaye, Centre Hospitalier Universitaire, Nîmes; J Duranteau, Hôpital de Bicêtre, Le Kremlin Bicêtre; B Francois, Hôpital Dupuytren, Limoges; N Smail, Hôpital Purpan, Toulouse; P Petit, Hôpital Edouard Herriot, Lyon; Germany: R Rossaint, Klinik für Anästhesie Universitätsklinikum Aachen, Aachen; HK van Aken, Universitätsklinikum Münster, Münster; G Hempelmann, Universitätsklinikum Giessen, Giessen. Israel: Y Kluger, Sourasky Medical Centre, Tel Aviv; A I Rivkind, Hadassah Medical Organisation, Jerusalem; G Shaked, Soroka Medical Centre, Beer Sheva; M Michaelson, Rambam Medical Centre, Haifa. Singapore: P Iau Tsau Choong, National University Hospital; A Yeo Wan Yan, Singapore General Hospital. Canada: SB Rizoli, Sunnybrook Health Sciences Centre, Toronto; SM Hameed, Foothills Medical Centre, Calgary. United Kingdom: GS Samra, The Royal London Hospital, London. Australia: GJ Dobb, Royal Perth Hospital, Perth.

The author group contributed significantly to the development of the protocol. In addition, the following contributed: USA: JA Asensio, Los Angeles, CA; W Biffl, Denver, CO; K Mattox and J Holcomb, Houston, TX; JH Patton, Detroit, MI; F Lewis, Pittsburgh, PA; M Lynn, Miami, FL; P O'Niel, Brooklyn, NY; JT Owings, Sacramento, CA; A Pietsman and S Tisherman, Pittsburgh, PA; TM Scalea, Baltimore, MD; and M Schreiber, Portland, OR.

## Figures and Tables

**Table 1:** Definitions of coagulopathy in the recent trauma literature

Laboratory parameters	Source
PT ≥1.5 N (0) 1.5–2 N (1) >2 N (2)	Mayo *et al*., 2004 [11]
PTT ≥1.5 N (0) 1.5–2 N (1) >2 N (2)	
Plt >100 (0) 50–100 (1) ≥50 (2)	
Fib >100 (0) 50–100 (1) ≥50 (2)	
Total: 0 mild, 1–3 moderate, 4–8 severe	
INR >1.4 or Plt <100,000 or both	Dutton *et al*., 2004 [9]
PT >18 seconds or PTT >60 seconds or TT >15 seconds	Brohi *et al*., 2003 [6]
PT >14 seconds or PTT >34 seconds	MacLeod *et al*., 2003 [7]
PT >15 seconds or PTT >45 seconds or Fib <100	Vaslef *et al*., 2002 [37]
PT or PTT twice normal	Cosgriff *et al*., 1997 [10]
PT or PTT >1.5–1.8 times control values	Stehling *et al*., 1996 [8]
Ongoing bleeding, oozing from cut surfaces, catheters, or mucous membranes	Lynn *et al*., 2002 [31]

Fib, fibrinogen; INR, international normalized ratio; N, normal; Plt, platelet; PT, prothrombin time; PTT, partial thromboplastin time; TT, thrombin time.

**Table 2:** Baseline characteristics of coagulopathic and non-coagulopathic patients

	Coagulopathic patients (*n *= 136)	Non-coagulopathic patients (*n *= 104)	*P *value
RBCs prior to trial drug, units (*n*)	8.4 ± 1.6 (134)	8.4 ± 1.4 (98)	1.00
FFP prior to trial drug, ml (*n*)	922 ± 627 (102)	95 ± 178 (88)	<0.001
Platelets prior to trial drug, ml (*n*)	126 ± 181 (128)	0 ± 0 (100)	<0.001
Cryoprecipitate prior to trial drug, ml (*n*)	28 ± 78 (135)	0 ± 0 (104)	<0.001

FFP after trial drug to 48 hours, ml (*n*)	1,596 ± 1,777 (102)	1,499 ± 1,927 (88)	0.72
Platelets after trial drug to 48 hours, ml (*n*)	289 ± 341 (127)	319 ± 432 (100)	0.57
Cryoprecipitate after trial drug to 48 hours, ml (*n*)	56 ± 151 (135)	33 ± 121 (104)	0.19

Injury severity score (*n*)	30 ± 13 (131)	26 ± 13 (104)	0.02
Temperature, °C (*n*)	35.1 ± 1.4 (92)	35.2 ± 1.5 (62)	0.68
Mean arterial pressure, mm Hg (*n*)	76 ± 22 (132)	74 ± 18 (101)	0.45
pH (*n*)	7.28 ± 0.11 (130)	7.24 ± 0.12 (101)	0.01
Hematocrit, percentage (*n*)	25 ± 8 (104)	28 ± 9 (83)	0.02
aPTT, seconds (*n*)	50 ± 25 (79)	54 ± 30 (51)	0.43
PT, seconds (*n*)	19 ± 6 (90)	21 ± 6 (58)	0.05
Platelet count, × 10^9 ^(*n*)	78 ± 45 (122)	70 ± 47 (98)	0.20
Fibrinogen, g/l (*n*)	1.3 ± 0.7 (90)	1.2 ± 1.2 (58)	0.57
48-hour mortality, *n *(percentage)	21 (15)	20 (19)	0.44

30-day mortality, *n *(percentage)	32 (24)	29 (28)	0.44

Data are mean ± standard deviation or number (percentage). aPTT, activated partial thromboplastin time; FFP, fresh frozen plasma; PT, prothrombin time; RBC, red blood cell.

**Table 3:** Baseline clinical and biological characteristics of coagulopathic patients in the placebo and rFVIIa groups

	Placebo group (*n *= 76)	rFVIIa group (*n *= 60)
Age (years)	34 ± 12	31 ± 11
Gender		
Men	63 (83%)	51 (85%)
Women	13 (17%)	9 (15%)
Type of trauma		
Penetrating	38 (50%)	35 (58%)
Blunt	38 (50%)	25 (42%)
Injury severity score	30 ± 13	30 ± 13
Number of body regions injured		
1	16 (21%)	13 (22%)
2–3	40 (53%)	29 (48%)
>3	18 (24%)	17 (28%)
Unknown	2 (3%)	1 (2%)
Systolic blood pressure (mm Hg)	103 ± 26	109 ± 31
Mean arterial pressure (mm Hg)	74 ± 22	78 ± 23
Body temperature (°C)	35.2 ± 1.6	35.6 ± 1.1
pH	7.27 ± 0.11	7.29 ± 0.10
Hematocrit (percentage)	23 ± 8	26 ± 7
aPTT (seconds)	54 ± 24	46 ± 26
PT (seconds)	20 ± 5	17 ± 7
Fibrinogen (g/l)	1.3 ± 0.8	1.4 ± 0.6
Platelets (× 10^9^)	70 ± 42	87 ± 47

Data are mean ± standard deviation or number (percentage). No significant statistical differences between groups were observed. aPTT, activated partial thromboplastin time; PT, prothrombin time; rFVIIa, recombinant activated factor VII.

**Table 4:** Transfusion requirements during the 48 hours after the first dose of trial drug in the placebo and rFVIIa groups

	Placebo group		rFVIIa group				
	*n*	Median (range)	*n*	Median (range)	Differences in medians	Estimated reduction: median of differences (95% CI)^a^	*P *value^b^

RBC (units)							
All patients	76	6.5 (0–41)	60	4.4 (0–39.9)	2.1	2.6 (0.1; 5.1)	0.02
Alive at 48 hours	65	6.6 (0–41)	48	2.9 (0–20)	3.7	3.5 (1.7; 5.7)	<0.001
FFP (ml)							
All patients	60	1,360 (0–6,912)	42	705 (0–9,000)	655	600 (0.00; 1,320)	0.04
Alive at 48 hours	54	1,400 (0–6,912)	35	660 (0–9,000)	740	800 (250.0; 1,420)	0.001
Platelets (ml)							
All patients	72	300 (0–1,500)	55	147 (0–1,200)	153	50 (0; 250)	0.09
Alive at 48 hours	62	300 (0–1,500)	46	100 (0–900)	200	50 (0; 250)	0.01

^a^The Hodges-Lehman shift with 95% CI. Patients who died within 48 hours were assigned the highest rank for the Wilcoxon-Mann-Whitney test and the Hodges-Lehman estimate. ^b^Two-sided Wilcoxon-Mann-Whitney rank sum test. CI, confidence interval; FFP, fresh frozen plasma; RBC, red blood cell; rFVIIa, recombinant activated factor VII.

**Table 5:** Baseline clinical and biological characteristics of coagulopathic patients who died early (within 48 hours)

	Placebo group	rFVIIa group
	(*n *= 10)	(*n *= 11)
Age (years)	39 ± 12	30 ± 9
Gender		
Men	7 (70%)	11 (100%)
Women	3 (30%)	0 (0%)
Type of trauma		
Penetrating	4 (40%)	8 (73%)
Blunt	6 (60%)	3 (27%)
Injury severity score	34 ± 13	37 ± 8
Number of body regions injured		
1	1 (10%)	2 (18%)
2–3	6 (60%)	7 (64%)
>3	3 (30%)	2 (18%)
Unknown	0 (0%)	0 (0%)
Systolic blood pressure (mm Hg)	87 ± 22	103 ± 34
Mean arterial pressure (mm Hg)	63 ± 17	68 ± 32
Body temperature (°C)	34.1 ± 2.5	35.2 ± 1.2
pH	7.28 ± 0.10	7.23 ± 0.09
Hematocrit (percentage)	26 ± 14	19 ± 4
aPTT (seconds)	93 ± 44	78 ± 32
PT (seconds)	25 ± 6	25 ± 7
Fibrinogen (g/l)	0.8 ± 0.3	0.7 ± 0.3
Platelets (× 10^9^)	42 ± 51	61 ± 35
Causes of death		
Exsanguination	7 (70%)	9 (82%)
Organ failure	3 (30%)	1(9%)
Cardiac injury	0 (0%)	1 (9%)

aPTT, activated partial thromboplastin time; PT, prothrombin time; rFVIIa, recombinant activated factor VII.

**Table 6:** Adverse events and clinical outcomes in the placebo and rFVIIa groups

All coagulopathic patients	Placebo group (*n *= 76)	rFVIIa group (*n *= 60)	*P *value
Thromboembolic events	3 (4%)	2 (3%)	1.00
Ventilator-free days	21 (0–29)	23 (0–29)	0.4
ICU-free days	12 (0–29)	18 (0–29)	0.3
Critical complication within 30 days			
ARDS	9 (12%)	1 (2%)	0.04
MOF	10 (13%)	2 (3%)	0.07
ARDS/MOF	15 (20%)	2 (3%)	0.004
Death	18 (24%)	14 (24%)	1.00
ARDS/MOF/death	25 (33%)	14 (23%)	0.26
Death within 48 hours	10 (13%)	11 (18%)	0.48
			

Coagulopathic patients surviving more than 48 hours	Placebo group (*n *= 66)	rFVIIa group (*n *= 49)	*P *value

Thromboembolic events	3 (5%)	1 (2%)	0.64
Ventilator-free days	23 (0–29)	25 (0–29)	0.08
ICU-free days	16 (0–29)	20 (0–29)	0.06
Critical complications within 30 days			
ARDS	9 (14%)	1 (2%)	0.04
MOF	8 (12%)	1 (2%)	0.08
ARDS/MOF	13 (20%)	1 (2%)	0.004
Death	8 (12%)	3 (6%)	0.4
ARDS/MOF/death	15 (23%)	3 (6%)	0.02

Data are median (range) or number (percentage). ARDS, acute respiratory distress syndrome; ICU, intensive care unit; MOF, multiple organ failure; rFVIIa, recombinant activated factor VII.
